# Complete Genome Sequence and Comparative Analysis of *Staphylococcus condimenti* DSM 11674, a Potential Starter Culture Isolated from Soy Sauce Mash

**DOI:** 10.3389/fbioe.2017.00056

**Published:** 2017-10-06

**Authors:** Huihui Dong, Jian Chen, Andrew K. Hastings, Lihua Guo, Beiwen Zheng

**Affiliations:** ^1^State Key Laboratory for Diagnosis and Treatment of Infectious Diseases, Collaborative Innovation Center for Diagnosis and Treatment of Infectious Diseases, The First Affiliated Hospital, College of Medicine, Zhejiang University, Hangzhou, China; ^2^Department of Internal Medicine, Section of Infectious Diseases, Yale University School of Medicine, New Haven, CT, United States; ^3^Intensive Care Unit, The First Affiliated Hospital, College of Medicine, Zhejiang University, Hangzhou, China

**Keywords:** *Staphylococcus condimenti*, complete genome, comparative genomic analysis, starter culture, fermentation

## Background

Coagulase-negative staphylococci (CNS) are key players in the majority of food fermentation ecosystems, which are commonly found in the production of fermented meat and milk products (Blaiotta et al., 2005; Resch et al., 2008). Strains of CNS have been implicated in exerting desirable effects as components of a fermentation flora, such as color formation, aroma development, and shelf-life enhancement, and may therefore have the potential for future application as starter cultures (Zell et al., 2008). *Staphylococcus condimenti* is one of the most prominent species and has the potential for use in starter cultures for the production of fermented sausage and cured ham (Zell et al., 2008). *S. condimenti* DSM 11674 was originally isolated from fermenting soy sauce mash and suggested to be a new species in 1998 (Probst et al., 1998). However, *S. condimenti* has been found in a few clinical samples (Argemi et al., 2015; Misawa et al., 2015). Therefore, some concerns have been raised with regard to the safety of this species for use in food production (Zell et al., 2008; Seitter et al., 2011a,b). To further understand the biochemical and genetic characteristics of DSM 11674 and advance the potential biotechnological applications of this strain, we constructed the complete genome sequence of *S. condimenti* DSM 11674.

## Materials and Methods

### Bacterial Strain and Biochemical Characterization

*Staphylococcus condimenti* DSM 11674 (= JCM 6074 = CIP 105760) was obtained from the Deutsche Sammlung von Mikroorganismen undZellkulturen. The isolate was identified by 16S rRNA sequencing. The sequence was then compared against NCBI database and EzTaxon-e database. To further explore its potential application in food fermentation, we calculated the nitrate reductase activity and catalase activity of *S. condimenti* DSM 11674 as described previously (Herrero et al., 1996; Miralles et al., 1996). Nitrite reductase activity was determined as described previously (Neubauer et al., 1999; Gotterup et al., 2007).

### Minimum Inhibitory Concentrations (MICs) and DNA Purification

Minimum inhibitory concentrations were established by the Vitek 2 Compact system with AST-GP67 card (bioMe’rieux, France). The MICs were interpreted according to Clinical and Laboratory Standards Institute (CLSI, 2016). Genomic DNA was extracted from 3-ml overnight cultures using a Gentra Puregene Yeast/Bact Kit (Qiagen, Hilden, Germany). Bacteria were treated with lysis buffer containing Proteinase K and RNaseA for 2 h at 65°C, and DNA purification was performed according to the manufacturer’s recommended protocols.

### Genome Sequencing and Assembly

The genome of *S. condimenti* DSM 11674 was sequenced on the PacBio RS II single-molecule real-time (SMRT) system. Raw sequence data were *de novo* assembled using the hierarchical genome-assembly process (HGAP) protocol (Chin et al., 2013) and RS HGAP Assembly 2.^1^

### Genome Annotation

The genome was annotated using the Rapid Annotation using Subsystem Technology server (Aziz et al., 2008) and the NCBI Prokaryotic Genome Annotation Pipeline. Ribosomal RNAs were detected by RNAmmer (Lagesen et al., 2007) and transfer RNAs by tRNAscan-SE (Lowe and Eddy, 1997). CRISPRFinder was used to screen for the presence of CRISPR elements (Grissa et al., 2007). Coding sequences were analyzed to detect toxin genes by using VirulenceFinder^2^ and by comparing the protein sequences using BLASTP with sequences in virulence factor database (Chen et al., 2005). The Antibiotic Resistance Genes Database was applied to classify antibiotic resistance genes (Liu and Pop, 2009).

### Comparative Genomic Analysis

The core genome alignment module in the rapid large-scale prokaryote pan genome analysis (Roary) pipeline was used to extract predicted coding regions from 21 complete *Staphylococci* genome sequences (Page et al., 2015). Core genes were defined as those present in all isolates with default parameters. Common and unique orthologous groups identified among the genomes were defined as previously described (Zheng et al., 2014). Full chromosome alignments were performed using progressive MAUVE (Darling et al., 2010).

## Results and Discussion

### Biochemical and Antimicrobial Characteristics

In our study, the strain of *S. condimenti* DSM 11674 has the highest capacity to reduce nitrate (13.67 mM nitrate reduced to nitrite per milligram of dry weight) and exhibits a high catalase activity compared to *Staphylococcus aureus* ATCC 25923 and the clinical isolate of *S. condimenti* CJ1628 (Figure S1A in Supplementary Material). Moreover, the strain of *S. condimenti* DSM 11674 exhibited the enhanced nitrite reductase activity when cultured with nitrite (2 mM) and nitrate (20 mM) under anaerobic condition (Table S1 in Supplementary Material). Antimicrobial susceptibility tests show that *S. condimenti* DSM 11674 is susceptible to all antibiotics tested, including amikacin, ampicillin/sulbactam, cefazolin, cefepime, ceftazidime, ceftriaxone, ciprofloxacin, ertapenem, gentamicin, imipenem, levofloxacin, tobramycin, and trimethoprim/sulfamethoxazole. These data are consistent with that of traditional starter culture *Staphylococcus carnosus* (Landeta et al., 2013) and indicate that *S. condimenti* is suitable as fermented meat starter.

### Genome Features

The complete circular chromosome was 2,659,676 bp with a G + C content of 34.7%. A total of 2,516 protein coding genes, 18 rRNA genes, 58 tRNA genes, 46 pseudogenes, and 2 CRISPR arrays were identified in the genome (Table S2 and Figure S2 in Supplementary Material).

### Comparison of Staphylococci Genomes

**Figure 1:**
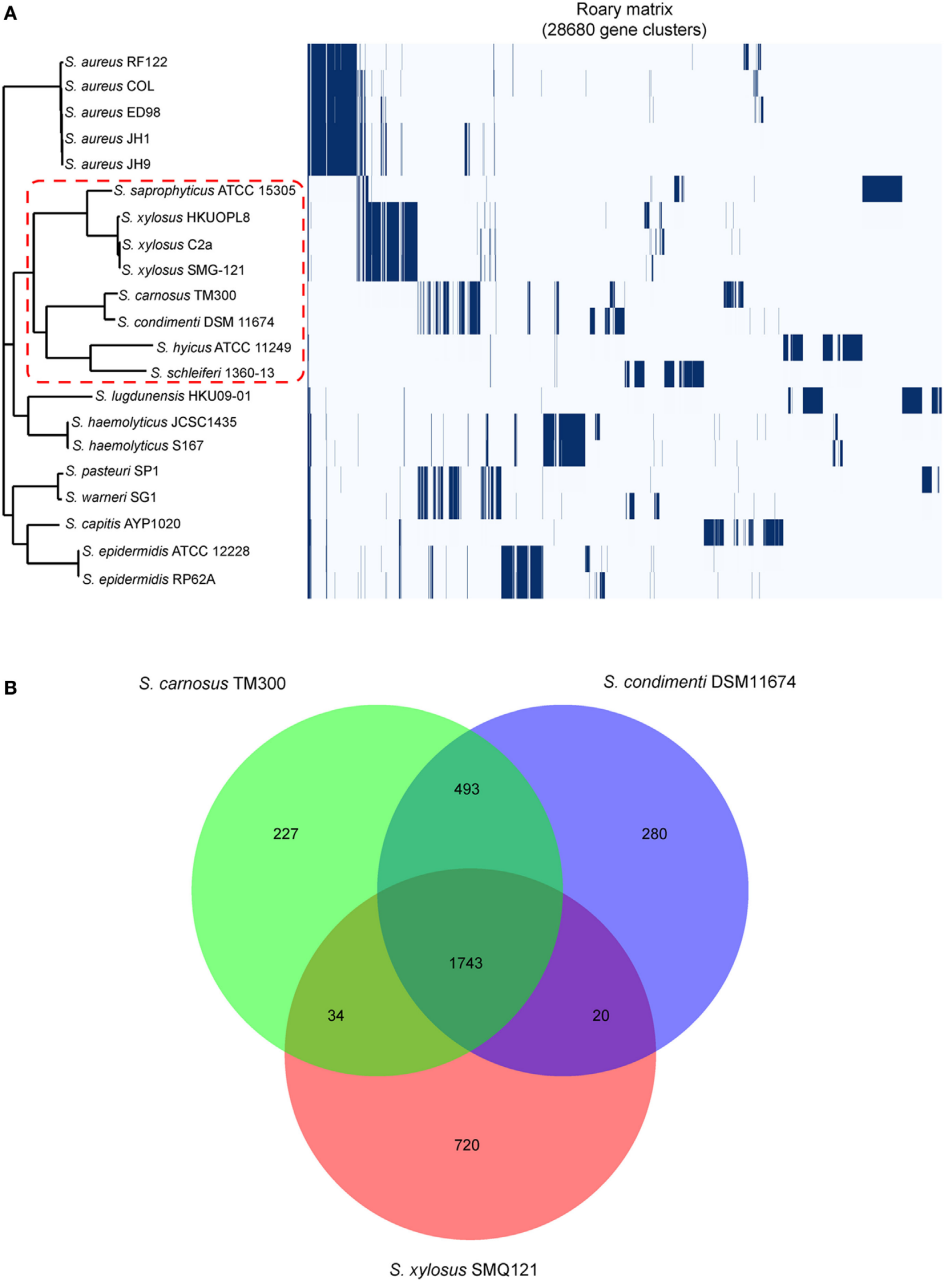
Genomic comparison of the *Staphylococcus condimenti* DSM 11674 with other staphylococci. **(A)** Phylogenetic tree based on all core gene sequences of 21 staphylococci complete genomes. Multiple sequence alignments of concatenated core gene sequences were calculated within Roary pipeline. The branch of the members of the *S. condimenti* is delimited by a red dash line. The isolates used in this study include *Staphylococcus schleiferi* 1360-13 (CP009470), *Staphylococcus epidermidis* RP62A (CP000029), *S. epidermidis* ATCC 12228 (AE015929), *Staphylococcus capitis* AYP1020 (CP007601), *Staphylococcus warneri* SG1 (CP003668), *Staphylococcus pasteuri* SP1 (CP004014), *Staphylococcus haemolyticus* S167 (CP013911), *S. haemolyticus* JCSC1435 (AP006716), *Staphylococcus lugdunensis* HKU09-01 (CP001837), *Staphylococcus hyicus* (CP008747), *S. condimenti* DSM 11674 (CP015114), *Staphylococcus carnosus* TM300 (AM295250), *Staphylococcus xylosus* SMG-121 (CP008724), *S. xylosus* C2a (LN554884), *S. xylosus* HKUOPL8 (CP007208.1), *Staphylococcus saprophyticus* ATCC 15305 (AP008934), *Staphylococcus aureus* RF122 (AJ938182), *S. aureus* COL (CP000046), *S. aureus* ED98 (CP001781), *S. aureus* JH1 (CP000736), and *S. aureus* JH9 (CP000703). **(B)** Core genome analysis of *S. condimenti* DSM 11674, *S. carnosus* TM300, and *S. xylosus* SMG-121. Numbers inside the Venn diagrams indicate the number of genes found to be shared among the indicated genomes.

A total of 28,680 gene clusters and 22 core genes were defined, and a phylogenetic tree was constructed based on the core gene alignment (Figure 1A; Table S3 in Supplementary Material). On this tree, *Staphylococcus saprophyticus* ATCC 15305, three *Staphylococcus xylosus* isolates, *S. carnosus* TM300, *Staphylococcus hyicus* ATCC11249, *Staphylococcus schleiferi* 1360-13, and *S. condimenti* DSM 11674 formed a monophyletic branch, providing strong evidence for the taxonomic relatedness of these isolates (Figure 1A). Of note, *S. condimenti* DSM 11674 has the closest relationship with *S. carnosus* TM300. On the basis of this, we further identified unique and shared gene content in *S. condimenti*, with commercial meat starter culture bacteria *S. carnosus* TM300 (Rosenstein et al., 2009) and *S. xylosus* SMG-121 (El Haddad et al., 2014), which are widely used in the food industry. A Venn diagram of the unique/shared gene content was generated with a custom R script using the VennDiagram package (Figure 1B). These three strains share 1,743 CDS in their genome. In addition, a noticeable overlap between DSM 11674 and TM300 became evident, and these two strains shared 493 orthologous CDS. Moreover, 280 CDS from the DSM 11674 genome were classified as unique. The MAUVE analysis revealed a significant portion of the genetic information has been conserved among DSM 11674 and TM300, as the majority of the local collinear blocks are shared by these two strains (Figure S3 in Supplementary Material).

### Fermentative Activity-Associated Genes

*In silico* analyses revealed that complete pathways involved in the reduction of nitrate to nitrite (nitrate reductase, WP_047131530) and further to ammonia (nitrite reductase, WP_047131535) were found in the genome of DSM 11674. Two catalases (WP_047130958, WP_047132101) were also identified in genome. These data are in agreement with our enzyme activity results and provide clues to explain the production of both nitrate reductase and catalase in DSM 11674. In addition, two l-lactate dehydrogenase (WP_047131934, WP_047132743) and two d-lactate dehydrogenase (WP_047132560, WP_047131604) were encoded, which match with the phenotypic trait that both l-lactate and d-lactate are produced in this strain (Probst et al., 1998). Interestingly, lactate dehydrogenase has been reported to play a role in the improvement of starter fermentative activity (Cheng et al., 2014). Therefore, these results indicated that DSM 11674 has strong potential for use as a novel starter culture.

### Salt-Dependent and Salt Acclimation Genes

During soy sauce mash fermentation, the DSM 11674 strain experiences significant osmotic stress. To explain the genetic determinants involved in the acclimation of this strain to high salt conditions, we identified several genes known to be important for survival under saline stress (Table 1). The strain contains six Na^+^/H^+^ antiporter subunits and seven monovalent cation/H^+^ antiporter subunits, which are homologs of the antiporter genes of *S. carnosus* TM300. Furthermore, we identified 15 additional salt-dependent and salt acclimation genes in the DSM 11674 strain. This high content of osmoprotective factors in the genome is consistent well with the ability of this species to grow readily in the presence of 15% NaCl (Probst et al., 1998).

**Table 1:** Summary of salt-dependent and salt acclimation genes in DSM 11674.

Gene name	Protein product	Length	Function of gene product
*nhaA*	WP_047132913	805	Na^+^/H^+^ antiporter subunit A
*nhaD*	WP_047132917	498	Na^+^/H^+^ antiporter subunit D
*nhaG*	WP_047132908	122	Na^+^/H^+^ antiporter subunit G
*nhaA*	WP_047132066	511	Na^+^/H^+^ antiporter subunit A
*nhaE*	WP_047132563	162	Na^+^/H^+^ antiporter subunit E
*nhaC*	WP_047131754	472	Na^+^/H^+^ antiporter subunit C
prk12573	WP_047132912	142	Monovalent cation/H^+^ antiporter subunit B
prk12651	WP_047132910	159	Monovalent cation/H^+^ antiporter subunit E
prk12600	WP_047132909	98	Monovalent cation/H^+^ antiporter subunit F
prk12646	WP_047132567	824	Monovalent cation/H^+^ antiporter subunit A
prk12574	WP_047132566	141	Monovalent cation/H^+^ antiporter subunit B
prk12663	WP_047132564	498	Monovalent cation/H^+^ antiporter subunit D
prk12657	WP_047132562	98	Monovalent cation/H^+^ antiporter subunit F
prk10429	WP_047131598	477	Melibiose/sodium symporter
*sdf*	WP_047132975	426	Na^+^/dicarboxylate symporter
*alsT*	WP_047131152	487	Na^+^/alanine symporter
*alsT*	WP_047131695	548	Na^+^/alanine symporter
*yuiF*	WP_047132905	438	Na^+^/proton antiporter
*nhaP*	WP_047131353	679	Na^+^/proton antiporter
*tyt1*	WP_047132938	443	Sodium-dependent transporter
*tcyP*	WP_047132269	461	Na^+^/dicarboxylate symporter
*putP*	WP_047133075	515	Na^+^/proline cotransporter PutP
*putP*	WP_047132835	513	Na^+^/proline cotransporter PutP
*arsB*	WP_047133077	497	Anion permease ArsB/NhaD
*yhaQ*	WP_047131485	299	Sodium ABC transporter ATP-binding protein
*natB*	WP_047131484	409	Sodium ABC transporter permease
*nhaC*	WP_047131472	437	Sodium/proton antiporter
*ccmA*	WP_047132811	296	Sodium ABC transporter ATP-binding protein

### Stress Response and Antimicrobial Resistance Genes

The DSM 11674 genome also possesses genes encoding an ATP synthase complex (WP_047132344-WP_047132350), which enables regulation of the internal pH and could confer the ability to adapt to stressful conditions (Cotter and Hill, 2003). Moreover, we identified two cold-shock protein-enconding (CspA and CspC) genes in the chromosome, which are involved in stress responses (Katzif et al., 2003). Also, the heat-shock regulon (*hrcA-grpE-dnaK-dnaJ* and *groESL*) (Singh et al., 2007; Rossi et al., 2017) and several other heat-shock protein encoding genes were found in DSM 11674. Finally, the screening of antimicrobial resistance genes revealed a putative β-lactamase encoding gene; this was consistent with susceptibility testing results. Thus, DSM 11674 strain shows technological characteristics that makes it a good candidate for biotechnical application.

In summary, this study reports the complete genome sequence of *S. condiment*, a bacterial strain that is potentially useful in a variety of food preparation applications. Genomics-based analysis of this functional staphylococci starter culture candidate revealed important insights into its metabolic capacities and niche adaptations. This is also the first comparative genome sequence analysis of staphylococci starter culture strains, revealing their core genome and pan genome. Finally, the biochemical and genetic characteristics of *S. condimenti* DSM 11674 revealed in this study are essential to generate further insights into the functional role of staphylococci in general and *S. condimenti* in particular during the food fermentation process.

## Ethics Statement

This article does not contain any studies with human participants or animals performed by any of the authors.

## Data Access

The complete genome sequence of *Staphylococcus condimenti* DSM 11674 has been deposited at DDBJ/EMBL/GenBank under the accession number CP015114.

## Author Contributions

BZ conceived and designed the research; HD and JC performed experiments and analyzed data; LG and AH analyzed data; BZ, HD, and AH wrote the manuscript; and all authors commented on the manuscript and approved the contents.

## Conflict of Interest Statement

The authors declare that the research was conducted in the absence of any commercial or financial relationships that could be construed as a potential conflict of interest.

## Footnotes

^1^https://github.com/PacificBiosciences/SMRT-Analysis.

^2^http://cge.cbs.dtu.dk/services/VirulenceFinder/.

## Supplementary Material

Figure S1 Biochemical characteristics of *Staphylococcus condimenti* DSM 11674. **(A)** Nitrate reductase activity of *S. condimenti* DSM 11674. **(B)** Catalase activity of *S. condimenti* DSM 11674. Enzyme activity was measured as spectrophotometric methods. *Staphylococcus aureus* ATCC 25923 and clinical isolate *S. condimenti* CJ1628 were used for comparison.

Figure S2 Genome atlas of *Staphylococcus condimenti* DSM 11674. The circles represent (from the outside to the inside): circle 1, reverse CDS (cyan); circle 2, forward CDS (yellow); circle 3, tRNAs (blue); circle 4, rRNAs (red); circle 5, GC plot; and circle 6, GC skew.

Figure S3 Genomic comparison of the *Staphylococcus condimenti* DSM 11674 with starter culture strains *Staphylococcus carnosus* TM300 and *Staphylococcus xylosus* SMQ121 by Mauve. Alignment is represented as local collinear blocks (LCBs) filled with a similarity plot. LCBs of conserved sequences among the strains are represented by rectangles of the same color. Connecting lines can be used to visualize synteny or rearrangement. LCBs positioned above or under the chromosome (black line) correspond to the forward and reverse orientation, respectively. The level of conservation is equivalent to the level of vertical color filling within the LCBs. Sequences not placed within an LCB are unique for the particular strain.

## References

[B1] Argemi X. Riegel P. Lavigne T. Lefebvre N. Grandpre N. Hansmann Y. (2015). Implementation of matrix-assisted laser desorption ionization-time of flight mass spectrometry in routine clinical laboratories improves identification of coagulase-negative staphylococci and reveals the pathogenic role of *Staphylococcus lugdunensis*. J. Clin. Microbiol. 53, 2030–2036. 10.1128/JCM.00177-15 25878345 PMC4473201

[B2] Aziz R. K. Bartels D. Best A. A. Dejongh M. Disz T. Edwards R. A. (2008). The RAST server: rapid annotations using subsystems technology. BMC Genomics 9:75 10.1186/1471-2164-9-75 18261238 PMC2265698

[B3] Blaiotta G. Casaburi A. Villani F. (2005). Identification and differentiation of *Staphylococcus carnosus* and *Staphylococcus simulans* by species-specific PCR assays of sodA genes. Syst. Appl. Microbiol. 28, 519–526. 10.1016/j.syapm.2005.03.007 16106559

[B4] Chen L. Yang J. Yu J. Yao Z. Sun L. Shen Y. (2005). VFDB: a reference database for bacterial virulence factors. Nucleic Acids Res. 33, D325–D328. 10.1093/nar/gki008 15608208 PMC539962

[B5] Cheng X. Dong Y. Su P. Xiao X. (2014). Improvement of the fermentative activity of lactic acid bacteria starter culture by the addition of Mn(2)(+). Appl. Biochem. Biotechnol. 174, 1752–1760. 10.1007/s12010-014-1156-z 25146195

[B6] Chin C. S. Alexander D. H. Marks P. Klammer A. A. Drake J. Heiner C. (2013). Nonhybrid, finished microbial genome assemblies from long-read SMRT sequencing data. Nat. Methods 10, 563–569. 10.1038/nmeth.2474 23644548

[B7] CLSI. (2016). Performance Standards for Antimicrobial Susceptibility Testing: Twenty-Six Informational Supplement M100-S26. Wayne, PA: Clinical and Laboratory Standards Institute.

[B8] Cotter P. D. Hill C. (2003). Surviving the acid test: responses of Gram-positive bacteria to low pH. Microbiol. Mol. Biol. Rev. 67, 429–453. 10.1128/MMBR.67.3.429-453.2003 12966143 PMC193868

[B9] Darling A. E. Mau B. Perna N. T. (2010). progressiveMauve: multiple genome alignment with gene gain, loss and rearrangement. PLoS ONE 5:e11147. 10.1371/journal.pone.0011147 20593022 PMC2892488

[B10] El Haddad L. Ben Abdallah N. Plante P. L. Dumaresq J. Katsarava R. Labrie S. (2014). Improving the safety of *Staphylococcus aureus* polyvalent phages by their production on a *Staphylococcus xylosus* strain. PLoS ONE 9:e102600. 10.1371/journal.pone.0102600 25061757 PMC4111496

[B11] Gotterup J. Olsen K. Knochel S. Tjener K. Stahnke L. H. Moller J. K. (2007). Relationship between nitrate/nitrite reductase activities in meat associated staphylococci and nitrosylmyoglobin formation in a cured meat model system. Int. J. Food Microbiol. 120, 303–310. 10.1016/j.ijfoodmicro.2007.08.034 17920151

[B12] Grissa I. Vergnaud G. Pourcel C. (2007). CRISPRFinder: a web tool to identify clustered regularly interspaced short palindromic repeats. Nucleic Acids Res. 35, W52–W57. 10.1093/nar/gkm360 17537822 PMC1933234

[B13] Herrero M. Mayo B. Gonzalez B. Suarez J. E. (1996). Evaluation of technologically important traits in lactic acid bacteria isolated from spontaneous fermentations. J. Appl. Bacteriol. 81, 565–570. 10.1111/j.1365-2672.1996.tb03548.x

[B14] Katzif S. Danavall D. Bowers S. Balthazar J. T. Shafer W. M. (2003). The major cold shock gene, cspA, is involved in the susceptibility of *Staphylococcus aureus* to an antimicrobial peptide of human cathepsin G. Infect. Immun. 71, 4304–4312. 10.1128/IAI.71.8.4304-4312.2003 12874306 PMC166043

[B15] Lagesen K. Hallin P. Rodland E. A. Staerfeldt H. H. Rognes T. Ussery D. W. (2007). RNAmmer: consistent and rapid annotation of ribosomal RNA genes. Nucleic Acids Res. 35, 3100–3108. 10.1093/nar/gkm160 17452365 PMC1888812

[B16] Landeta G. Curiel J. A. Carrascosa A. V. Munoz R. De Las Rivas B. (2013). Characterization of coagulase-negative staphylococci isolated from Spanish dry cured meat products. Meat Sci. 93, 387–396. 10.1016/j.meatsci.2012.09.019 23273441

[B17] Liu B. Pop M. (2009). ARDB – antibiotic resistance genes database. Nucleic Acids Res. 37, D443–D447. 10.1093/nar/gkn656 18832362 PMC2686595

[B18] Lowe T. M. Eddy S. R. (1997). tRNAscan-SE: a program for improved detection of transfer RNA genes in genomic sequence. Nucleic Acids Res. 25, 955–964. 10.1093/nar/25.5.0955 9023104 PMC146525

[B19] Miralles M. C. Flores J. Perezmartinez G. (1996). Biochemical tests for the selection of *Staphylococcus* strains as potential meat starter cultures. Food Microbiol. 13, 227–236. 10.1006/fmic.1996.0028

[B20] Misawa Y. Yoshida A. Okugawa S. Moriya K. (2015). First reported case of *Staphylococcus condimenti* infection associated with catheter-related bacteraemia. New Microbes New Infect. 3, 18–20. 10.1016/j.nmni.2014.10.002 25755886 PMC4337941

[B21] Neubauer H. Pantel I. Gotz F. (1999). Molecular characterization of the nitrite-reducing system of *Staphylococcus carnosus*. J. Bacteriol. 181, 1481–1488. 10049379 10.1128/jb.181.5.1481-1488.1999 PMC93537

[B22] Page A. J. Cummins C. A. Hunt M. Wong V. K. Reuter S. Holden M. T. (2015). Roary: rapid large-scale prokaryote pan genome analysis. Bioinformatics 31, 3691–3693. 10.1093/bioinformatics/btv421 26198102 PMC4817141

[B23] Probst A. J. Hertel C. Richter L. Wassill L. Ludwig W. Hammes W. P. (1998). *Staphylococcus condimenti* sp. nov., from soy sauce mash, and *Staphylococcus carnosus* (Schleifer and Fischer 1982) subsp. utilis subsp. nov. Int. J. Syst. Bacteriol. 48(Pt 3), 651–658. 10.1099/00207713-48-3-651 9734019

[B24] Resch M. Nagel V. Hertel C. (2008). Antibiotic resistance of coagulase-negative staphylococci associated with food and used in starter cultures. Int. J. Food Microbiol. 127, 99–104. 10.1016/j.ijfoodmicro.2008.06.013 18625535

[B25] Rosenstein R. Nerz C. Biswas L. Resch A. Raddatz G. Schuster S. C. (2009). Genome analysis of the meat starter culture bacterium *Staphylococcus carnosus* TM300. Appl. Environ. Microbiol. 75, 811–822. 10.1128/AEM.01982-08 19060169 PMC2632126

[B26] Rossi C. C. De Oliveira L. L. De Carvalho Rodrigues D. Urmenyi T. P. Laport M. S. Giambiagi-Demarval M. (2017). Expression of the stress-response regulators CtsR and HrcA in the uropathogen *Staphylococcus saprophyticus* during heat shock. Antonie Van Leeuwenhoek 110, 1105–1111. 10.1007/s10482-017-0881-z 28455762

[B27] Seitter M. Geng B. Hertel C. (2011a). Binding to extracellular matrix proteins and formation of biogenic amines by food-associated coagulase-negative staphylococci. Int. J. Food Microbiol. 145, 483–487. 10.1016/j.ijfoodmicro.2011.01.026 21333369

[B28] Seitter M. Nerz C. Rosenstein R. Gotz F. Hertel C. (2011b). DNA microarray based detection of genes involved in safety and technologically relevant properties of food associated coagulase-negative staphylococci. Int. J. Food Microbiol. 145, 449–458. 10.1016/j.ijfoodmicro.2011.01.021 21329998

[B29] Singh V. K. Utaida S. Jackson L. S. Jayaswal R. K. Wilkinson B. J. Chamberlain N. R. (2007). Role for dnaK locus in tolerance of multiple stresses in *Staphylococcus aureus*. Microbiology 153, 3162–3173. 10.1099/mic.0.2007/009506-0 17768259

[B30] Zell C. Resch M. Rosenstein R. Albrecht T. Hertel C. Gotz F. (2008). Characterization of toxin production of coagulase-negative staphylococci isolated from food and starter cultures. Int. J. Food Microbiol. 127, 246–251. 10.1016/j.ijfoodmicro.2008.07.016 18752861

[B31] Zheng B. Zhang F. Chai L. Yu G. Shu F. Wang Z. (2014). Permanent draft genome sequence of *Geobacillus thermocatenulatus* strain GS-1. Mar. Genomics 18PB, 129–131. 10.1016/j.margen.2014.09.005 25280889

