# Supplementary material for: Complete Genome Sequence and Comparative Analysis of Staphylococcus condimenti DSM 11674, a Potential Starter Culture Isolated from Soy Sauce Mash
Source: Front Bioeng Biotechnol. 2017 Oct 6;5:56. doi: 10.3389/fbioe.2017.00056 (PMC5635325; doi:10.3389/fbioe.2017.00056)
Supplement: Supplementary file 1 [file Table_1.DOCX]

**Table S1.** Nitrite reductase activities of *S. condimenti* DSM 11674

|  | Activity (nmol min^-1^ ml^-1^) | | |
| --- | --- | --- | --- |
|  | + NO_3_- (20 mM) | + NO_2_- (2 mM) | - |
| Aerobic | 0 | 0 | 0 |
| Anaerobic | 36 | 15 | 7 |
