# Supplementary material for: Complete Genome Sequence and Comparative Analysis of Staphylococcus condimenti DSM 11674, a Potential Starter Culture Isolated from Soy Sauce Mash
Source: Front Bioeng Biotechnol. 2017 Oct 6;5:56. doi: 10.3389/fbioe.2017.00056 (PMC5635325; doi:10.3389/fbioe.2017.00056)
Supplement: Supplementary file 2 [file Table_2.DOCX]

**Table S2.** General genome features of *S. condimenti* DSM 11674

| Attributes | Value |
| --- | --- |
| Genomic size (bp) | 2,659,676 |
| Contig numbers | 1 |
| Coverage | 262 |
| GC content (%) | 34.7 |
| Number of Coding Sequences | 2,516 |
| Number of Subsystmes | 389 |
| rRNAs | 18 |
| tRNAs | 58 |
| Pseudogenes | 46 |
| CRISPRs | 2 |
| GenBank accession no. | CP015114 |
