# Supplementary figures and images for: Complete Genome Sequence and Comparative Analysis of Staphylococcus condimenti DSM 11674, a Potential Starter Culture Isolated from Soy Sauce Mash
Source: Front Bioeng Biotechnol. 2017 Oct 6;5:56. doi: 10.3389/fbioe.2017.00056 (PMC5635325; doi:10.3389/fbioe.2017.00056)

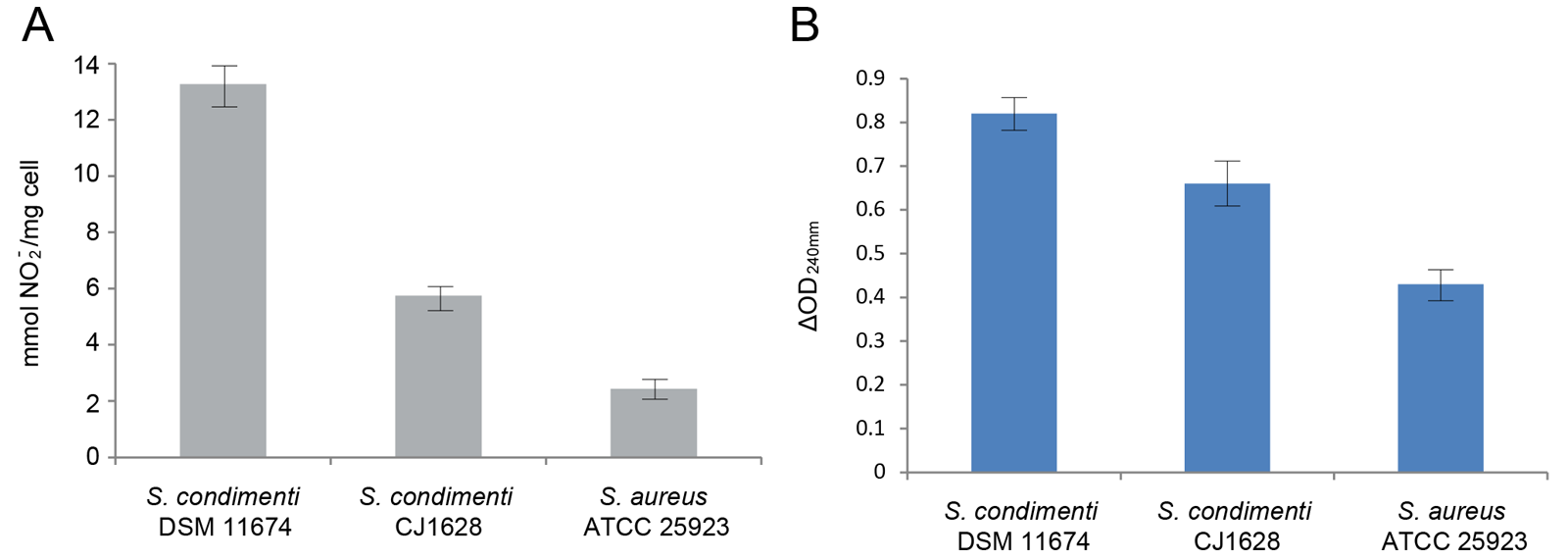

Supplement: Figure S1 — Biochemical characteristics of Staphylococcus condimenti DSM 11674. (A) Nitrate reductase activity of S. condimenti DSM 11674. (B) Catalase activity of S. condimenti DSM 11674. Enzyme activity was measured as spectrophotometric methods. Staphylococcus aureus ATCC 25923 and clinical isolate S. condimenti CJ1628 were used for comparison. [file Image_1.TIF]

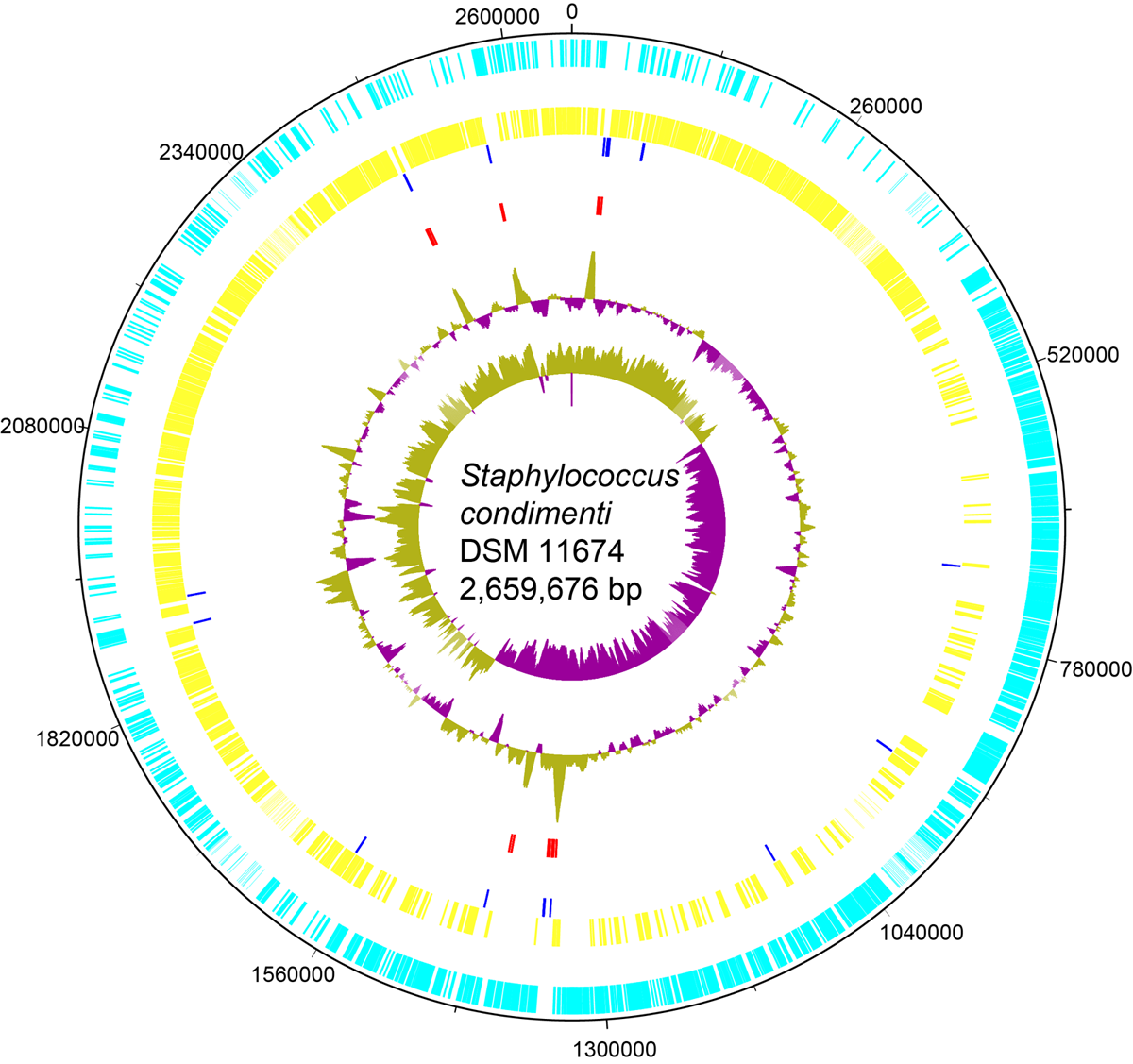

Supplement: Figure S2 — Genome atlas of Staphylococcus condimenti DSM 11674. The circles represent (from the outside to the inside): circle 1, reverse CDS (cyan); circle 2, forward CDS (yellow); circle 3, tRNAs (blue); circle 4, rRNAs (red); circle 5, GC plot; and circle 6, GC skew. [file Image_2.TIF]

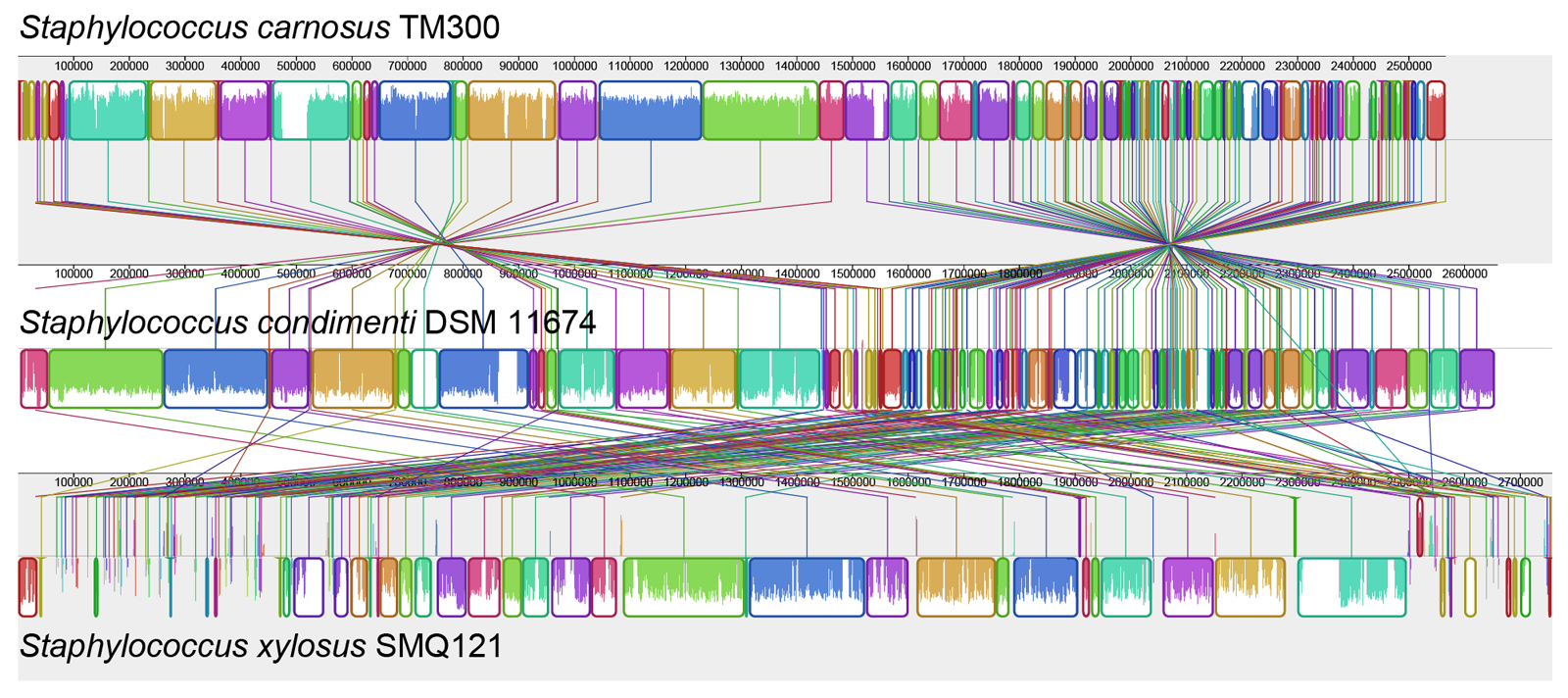

Supplement: Figure S3 — Genomic comparison of the Staphylococcus condimenti DSM 11674 with starter culture strains Staphylococcus carnosus TM300 and Staphylococcus xylosus SMQ121 by Mauve. Alignment is represented as local collinear blocks (LCBs) filled with a similarity plot. LCBs of conserved sequences among the strains are represented by rectangles of the same color. Connecting lines can be used to visualize synteny or rearrangement. LCBs positioned above or under the chromosome (black line) correspond to the forward and reverse orientation, respectively. The level of conservation is equivalent to the level of vertical color filling within the LCBs. Sequences not placed within an LCB are unique for the particular strain. [file Image_3.TIF]
